# The use of deep learning on endoscopic images to assess the response of rectal cancer after chemoradiation

**DOI:** 10.1007/s00464-021-08685-7

**Published:** 2021-10-12

**Authors:** Hester E. Haak, Xinpei Gao, Monique Maas, Selam Waktola, Sean Benson, Regina G. H. Beets-Tan, Geerard L. Beets, Monique van Leerdam, Jarno Melenhorst

**Affiliations:** 1 Department of Surgery, Netherlands Cancer Institute—Antoni Van Leeuwenhoek, Amsterdam, The Netherlands; 2 GROW School for Oncology and Developmental Biology—Maastricht University, Maastricht, The Netherlands; 3 Department of Radiology, Netherlands Cancer Institute-Antoni Van Leeuwenhoek, Amsterdam, The Netherlands; 4 Department of Gastroenterology, Netherlands Cancer Institute-Antoni Van Leeuwenhoek, Amsterdam, The Netherlands; 5 Department of Surgery, Maastricht University Medical Centre, Postbox 5800, 6202 AZ Maastricht, The Netherlands

**Keywords:** Rectal cancer, Deep learning, Response evaluation, Organ preservation, Watch-and-wait approach, Artificial intelligence

## Abstract

**Background:**

Accurate response evaluation is necessary to select complete responders (CRs) for a watch-and-wait approach. Deep learning may aid in this process, but so far has never been evaluated for this purpose. The aim was to evaluate the accuracy to assess response with deep learning methods based on endoscopic images in rectal cancer patients after neoadjuvant therapy.

**Methods:**

Rectal cancer patients diagnosed between January 2012 and December 2015 and treated with neoadjuvant (chemo)radiotherapy were retrospectively selected from a single institute. All patients underwent flexible endoscopy for response evaluation. Diagnostic performance (accuracy, area under the receiver operator characteristics curve (AUC), positive- and negative predictive values, sensitivities and specificities) of different open accessible deep learning networks was calculated. Reference standard was histology after surgery, or long-term outcome (>2 years of follow-up) in a watch-and-wait policy.

**Results:**

226 patients were included for the study (117(52%) were non-CRs; 109(48%) were CRs). The accuracy, AUC, positive- and negative predictive values, sensitivity and specificity of the different models varied from 0.67–0.75%, 0.76–0.83%, 67–74%, 70–78%, 68–79% to 66–75%, respectively. Overall, EfficientNet-B2 was the most successful model with the highest diagnostic performance.

**Conclusions:**

This pilot study shows that deep learning has a modest accuracy (AUCs 0.76-0.83). This is not accurate enough for clinical decision making, and lower than what is generally reported by experienced endoscopists. Deep learning models can however be further improved and may become useful to assist endoscopists in evaluating the response. More well-designed prospective studies are required.

**Supplementary Information:**

The online version contains supplementary material available at 10.1007/s00464-021-08685-7.

Rectal cancer patients treated with neoadjuvant (chemo)radiotherapy (CRT) usually undergo revaluation 6–10 weeks after the end of radiotherapy to evaluate therapy response. With an increasing interest in organ preservation, an additional goal of response evaluation is to identify a possible (near) complete response (CR). A combination of three modality assessment, digital rectal examination (DRE), endoscopy and MRI with diffusion-weighted imaging (DWI), has been shown to have the highest accuracy to identify a CR [1]. Many studies have addressed the value of MRI, while few studies have focused on endoscopy. Those which did evaluate the diagnostic value of endoscopy showed that it outperformed MRI in assessing the response [1, 2]. The majority of patients with a luminal CR (>70%) can be identified with endoscopy and a flat white scar is the most predictive feature to identify a CR [2]. However, 26 – 36% of the patients show other subtle morphological abnormalities, such as small or large flat ulcers, irregular tissue or residual adenomas, which are more difficult to interpret, leading to a considerable risk of missing residual disease or CRs [1–3]. New endoscopic techniques with computer aided diagnosis (CAD) using advanced imaging as narrow band imaging (NBI) or magnifying chromoendoscopy are designed to aid endoscopists in evaluating the histology of mucosal lesions, for example, by predicting submucosal invasions in advanced adenoma [4]. However, these techniques have not been studied in response assessment, and are limited due to the variability in diagnostic performance [5]. Other advances, in the field of artificial intelligence, in particular deep learning, may have potential to improve the endoscopic diagnostic accuracy [5, 6]. Deep learning neural networks use many layers to automatically extract features. Automated methods such as deep learning are capable of analyzing large amounts of images at much faster rates than a human. It has already been shown to be effective in detecting small esophageal cancer lesions [7, 8] or (benign) polyps in colon cancer [9–11] on endoscopy. The aim of this pilot study is to evaluate the feasibility and accuracy of deep learning methods based on endoscopic images and clinical variables for the response evaluation of rectal cancer patients treated with neoadjuvant therapy.

## Materials and methods

### Study design

The study cohort was retrospectively selected from a single institute database between January 2012 and December 2015. Informed consent was waived by the local institutional review board. Patients were included if they had (1) primary rectal cancer, (2) neoadjuvant long-course CRT or short course radiotherapy both followed by a waiting interval for downsizing, and (3) restaging endoscopic images available. Endoscopic restaging was routinely performed to assess the luminal response after neoadjuvant therapy. When residual disease was present at the response evaluation patients were referred for a total mesorectal excision (TME). When there was evidence of a clinical CR, patients were followed in a prospective watch-and-wait (W&W) study, approved by the local institutional review board and registered on clinicaltrials.gov (NCT00939666 and NCT02278653). A clinical CR, as described in Maas et al. [12], consisted of no palpable tumor on DRE, white scar with no residual mass, ulcer or irregularity on endoscopy, and substantial downsizing with residual homogeneous fibrosis on T2-weighted imaging (T2W) without high signal on diffusion-weighted imaging (DWI). Patients were excluded if they were: (1) lost to follow-up (FU), (2) refused surgery despite residual disease, or (3) maximum FU < 2 years when followed in a W&W program.

### Endoscopy

All patients underwent flexible endoscopy (EPK-I video processor, Pentax Medical Netherlands, Uithoorn, the Netherlands) after neoadjuvant therapy to evaluate the luminal response. All patients received a rectal phosphate enema as a bowel preparation prior to endoscopy. Endoscopy was performed with standard white light imaging and the images (resolution of 768 × 576 pixels and 300 × 300 dpi) were digitally stored afterward.

## Predictive models

### Model based on clinical variables

From the total of 226 patients, 70% (*n* = 158) were randomly allocated to a training/validation subset, 30% (*n* = 68) to a test subset, stratifying for CR and non-CR status. Three predictive models namely feedforward neural network (FFN), support vector machine (SVM) and logistic regression were built based on six clinical variables (age, sex, clinical T-stage, clinical N-stage, neoadjuvant treatment, and time between restaging endoscopy and surgery).

To reduce overfitting and improve the accuracy of the predictive models, the SelectKBest feature selection technique [13] was used to choose the best predicting clinical variables for the outcome (CR or non-CR). This technique scores all the features and then selects the optimal features according to the top highest scores. The top three selected clinical features (clinical N-stage, neoadjuvant treatment, and time between restaging endoscopy and surgery) were found to be optimum to train the models [14]. Performance of the clinical model was further assessed with the outcome measurements AUC, accuracy, precision, sensitivity and the F1-score. The F1-score is calculated as follows: F1Score = 2*((precision*sensitivity)/(precision + sensitivity).

During training, fivefold cross-validation was used on 70% of the training and validation set. The area under the curve (AUC) was calculated to assess model performance, where the loss function is minimized. During testing, bootstrapping calculated the model performance (AUC) of 500 randomly selected samples (with replacement) of the test subset. Mean AUC and the standard deviation of these 500 iterations were calculated to measure the model performance and the variability, respectively.

### Deep learning based on endoscopy

#### Image preprocessing

**Fig. 1:**
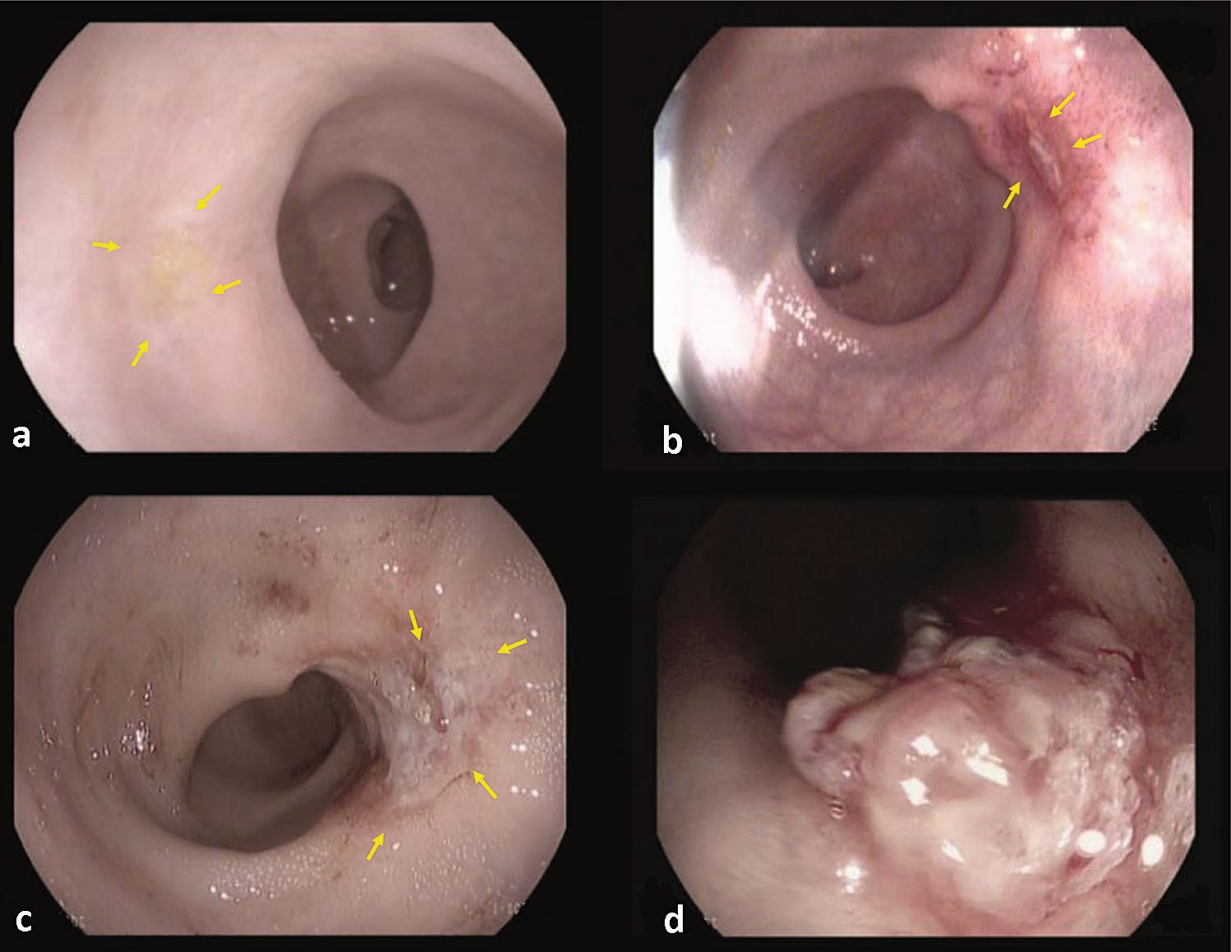
Example of evident and doubtful complete responders and non-complete responders. Evident complete response with a typical white scar (yellow arrows) (**a**), doubtful response with a small ulcer (yellow arrows) (**b**), doubtful response with a small-medium sized ulcer (yellow arrows) (**c**), and evident incomplete response with a tumor mass (**d**) (Color figure online)

**Fig. 2:**
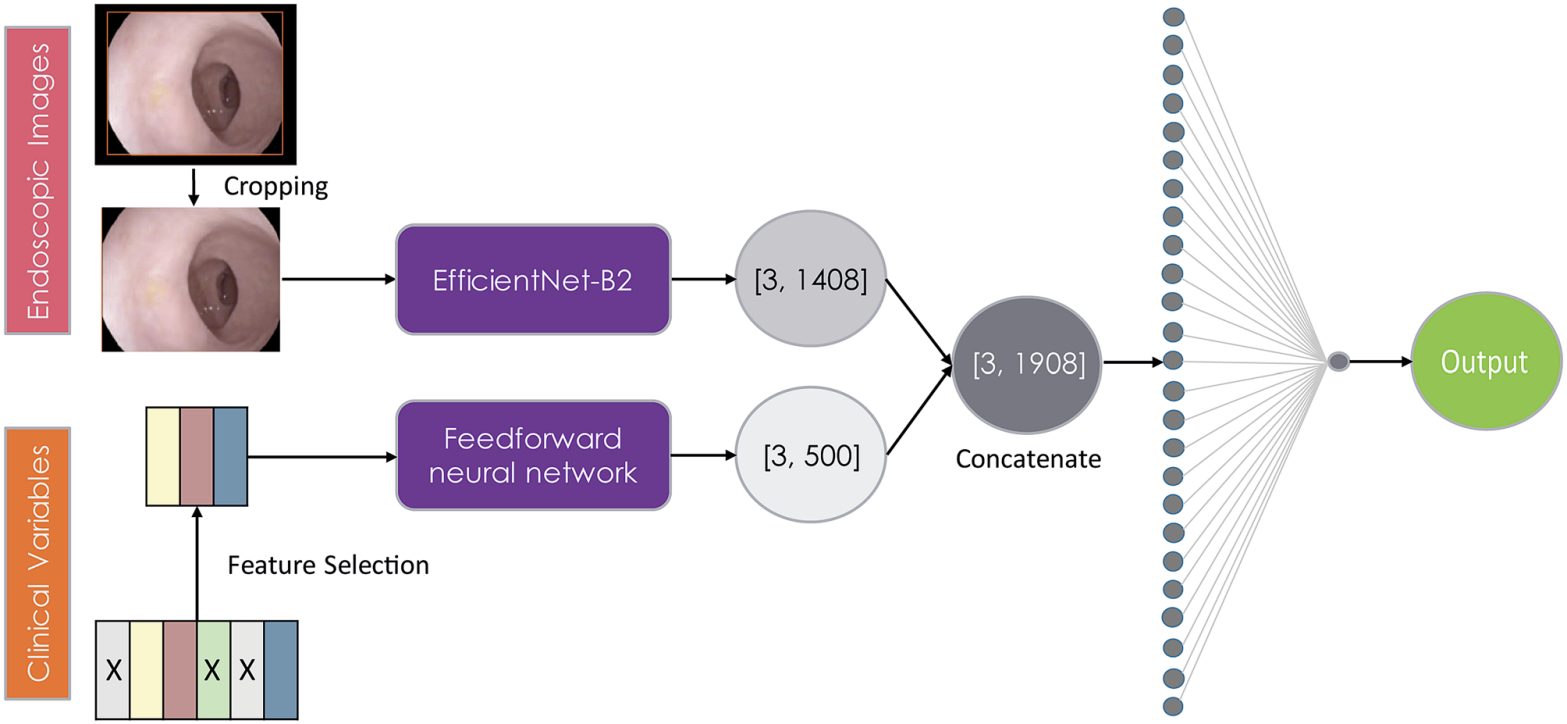
Overview of the combined model architecture. [1408] Represents the last channels in EfficientNet-B2. [500] Represents the number of neurons in feedforward neural network based on three selected clinical features

A total of 731 endoscopic images were used which were split into training, validation and testing set, with the portion 7:3 resulting in 512 training/validation images and 219 test images. Since the number of available images were limited, fivefold cross-validation was used to evaluate all the deep learning models. All endoscopic images belonging to the same patient were included in the same set. The median number of images per patient was 3 (range 2–7). The training set was used for the optimization of the weight of the neural network by the training process; the validation set was used to adjust the hyper-parameters (learning rate, number of epochs and size of mini batches) and the test set was independent from the training procedure, to test the final result of the neural network. The neural networks are trained using an optimization process that requires a loss function to calculate the error in the model. During training, if the prediction matches with the actual results the values of the loss function will be lower. The results of the independent test set will be presented. Figure 1 shows examples of the endoscopic images of CRs and non-CRs. The endoscopic images were first preprocessed to focus on the important features of the image (Fig. 2). Preprocessing consisted of cutting the black margin of the images followed by cropping out the central region of the image. The images were also resized based on model image input size, rescaled and normalized. We applied a data augmentation procedure to increase the number of images used for the training set and to avoid overfitting [15]. The images for training were expanded by rotation, flipping, shearing and zooming of the original images, resulting in 4 additional images per patient. The number of CRs in the training/validation and test set were 76 and 33 patients, respectively.

#### Convolutional neural networks (CNN)

To develop an accurate deep learning algorithm large datasets are needed using a vast amount of data. An alternative way is to use transfer learning via ImageNet [16], which is an online accessible tool and re-uses networks which were trained by an enormous amount of natural images. Fine-tuning existing CNN models that have been pre-trained with a large set of labeled natural images are common to use as a transfer learning technique. The re-used network can be performed on medical data and show promising results [17, 18]. Specifically, models trained on the ImageNet dataset (~ 1.2 million training RGB images) could be suitable to apply transfer training learning while training endoscopic images where the available dataset is limited [17]. To improve the output of CNN, a classical method is used to increase the number of layers of CNN, which will also lead to the increase of time to compute and the difficulty to converge. As the number of layers increases, when the gradient is back propagated, multiple times of multiplication will make the gradient unstable, which is called a gradient explode or vanish problem. To improve this, novel structures are used. “ResNet” have shortcut connections between layers, and the output of previous layer will be added to the input of later layers [19]. ResNet’s design is able to stabilize gradient and relieve the gradient explosion or vanishing problem. “DenseNet” has a similar concept with ResNet, and connects all the output of previous layers to the later layer by concatenating [20]. DenseNet can also relieve the gradient explode or vanish problem and needs less computational time and memory compared to ResNet. Inception adds multiscale convolution modules in parallel, collects multiscale feature maps and concatenate, to increase the ability to learn feature representation [21]. Inception avoids the problem of adding too many layers. “InceptionResNet” combines the basic module of Inception and ResNet to achieve the advantages of both [22]. “Xception” is an improved version of Inception [23]. It replaces the convolution modules in Inception to depth wise separable convolutions, separating completely the relevance of channels. “MobileNet” separates convolution into depth wise and pointwise convolutions, compresses the network and also keeps the accuracy level [24]. “EfficientNet” is one of the recent convolutional neural network architectures that achieve much better accuracy and efficiency as compared to the previous ConvNets. It uses a new scaling method that uniformly scales all dimensions of depth, width, and resolution to obtain a family of deep learning models [25]. In our study, we test and compared several CNNs including Xception, MobileNetV2, DenseNet121, ResNet50, InceptionV3, InceptionResNetV2 and EfficientNet-B2. They are mostly models with top results in natural object recognition in ImageNet Large Scale Visual Recognition Challenge(ILSVRC) competition [26]. The CNNs were trained with two 4 GB K2 Nvidia Graphics Processing Unit (GPU)s. The optimizer was Adam with the learning rate 1e-4. To find the best optimizer, we also tried SGD (stochastic gradient descent), RMSprop, and the learning rate was adjusted from 1e-3 to 1e-5. All the layers of the models were trained; and reducing the number of layers trained did not improve the performance. We also tried to change the training scheme, such as first training the bottom layer, then all the layers, which did not make a difference with current methods. The initialization weights of the models were the weight trained by ImageNet, the results became much worse with random initialized weights.

### Combined model

Deep learning models in medical applications are increasingly combining contextual data from electronic health records and pixel data, because the clinical context is often crucial in diagnostic decisions [27]. Hence, in the present study the clinical features are combined with endoscopic imaging features to improve the performance of the deep learning models and provide more clinically relevant models. FFN was chosen to combine the selected clinical features (clinical N-stage, neoadjuvant treatment, and time between restaging endoscopy and surgery), in which it was the best performing model from the models constructed based on clinical variables. The late fusion technique [28] is used to train the combined models where the deep learning models extract features from the endoscopic images and the FFN part extracts features from the selected clinical variables. The combined model architecture is presented in Fig. 2.

## Reference standard

The outcome of the deep learning method was compared with the reference standard: non-CRs or CRs. In patients who were operated, the histopathological staging of the surgical resection specimen provided the reference standard, and in W&W patients follow-up provided the reference standard. Non-CRs were either defined as patients who had residual luminal disease at histopathology (yT1-4) after resection, or W&W patients who developed a local regrowth (LR) during follow-up. CRs were defined as patients who had a pathological complete response (pCR) according histopathology (ypT0) or W&W patients with a sustained clinical CR after at least 2 years, as the vast majority of LRs occur within the first two years of FU [29, 30]. Because this study focused on the luminal response assessment, nodal stage was not included.

### Statistical analysis

Statistical analyses were performed using IBM SPSS Statistics version 22.0 (IBM Corporation 2013, Armonk, NY). For this pilot study no formal sample size calculation was made. Nominal data are presented as absolute frequencies and values and continuous data as median numbers with interquartile range (IQR). Baseline characteristics were compared between patients with and without a CR during FU. Differences were tested for significance with the χ^2^ test for the comparison of proportions and the use of Mann–Whitney U-test for comparison of the medians. The diagnostic performance of the deep learning models was calculated by use of the following parameters: accuracy, area under the receiver operator characteristics curve (AUC), sensitivity, specificity, positive predictive value (PPV), and negative predictive value (NPV) with 95% confidence intervals (CIs). The diagnostic performance of all the parameters was calculated according the binary outcome (CR or no-CR), and CR was the positive outcome measure.

## Results

### Demographics

238 patients were eligible for the study, of which 12 patients were excluded for the following reasons: W&W FU less than 2 years (*n* = 2), refused surgery despite residual disease (*n* = 5), lost to FU (*n* = 2) and missing values (*n* = 3). A total of *n* = 226 patients were included in the analysis. Demographics of all patients are shown in Table 1. Median age was 65 (58–73) years and 153 (68%) of the patients were male. 206 (90%) of the patients received neoadjuvant CRT, the remaining 20 (10%) patients had short course radiotherapy with a prolonged waiting interval. In total, 117 (52%) of 226 patients had residual disease: 94 patients after immediate surgery and 23 patients in the W&W program who developed a regrowth (16 ypT1, 41 ypT2, 56 ypT3 and 4 ypT4). 109 (48%) of 226 patients were CRs: 19 with ypT0 after TME surgery, 85 W&W patients with a sustained ycT0 with a median FU of 53 months (26–69), and 5 W&W patients who underwent surgery for a suspected regrowth but did have a ypT0.

**Table 1:** Patient characteristics of the total cohort and with and without a complete response during follow-up

Variables	All (*n* = 226)	Non-CR (*n* = 117)	CR (*n* = 109)	*P*
Age, median (IQR), year	65 (58–73)	65 (58–74)	66 (59–73)	0.952
Sex, *n* (%)				
Male	153 (68)	78 (67)	75 (69)	0.731
Female	73 (32)	39 (33)	34 (31)	
Clinical T-stage, *n*(%)				
1–2	49 (22)	21 (18)	28 (26)	0.095
3	161 (71)	84 (72)	77 (70)	
4	16 (7)	12 (10)	4 (4)	
Clinical N-stage, *n*(%)				
0	54 (24)	26 (22)	28 (26)	0.038
1	64 (28)	26 (22)	38 (35)	
2	108 (48)	65 (56)	43 (39)	
Distance anal verge, *n*(%)				
≤ 5 cm	165 (73)	80 (68)	85 (78)	0.042
≥ 5 cm	61 (27)	37 (32)	24 (22)	
Neoadjuvant treatment, *n*(%)				
5 × 5 Gy + prolonged waiting interval	20 (10)	16 (14)	4 (4)	<0.001
CRT	206 (90)	101 (86)	105 (96)	
Adjuvant chemotherapy, *n*(%)				
Yes	41 (18)	22 (19)	19 (17)	0.227
No	185 (82)	95 (81)	90 (83)	
Time between last radiotherapy and endoscopy, median (IQR), weeks	10 (8–15)	8 (8–12)	12 (9–18)	<0.001
Time between restaging endoscopy and surgery, median (IQR), weeks	5 (2–10)	4 (2–12)	6 (3–10)	0.359
Final treatment, *n*(%)				
W&W	113 (50)	23 (20)	90 (83)	<0.001
Immediate surgery	113 (50)	94 (80)	19 (17)	

*CR* Complete response, *no-CR* No complete response, *P* p-value, *IQR* Interquartile range, *Gy* Gray, *CRT* Chemoradiation, *W&W* Watch-and-wait

### Performance of the models

In this section, we show the automatically generated results of models constructed based on clinical features, endoscopic images and combined (endoscopic images and clinical features) models of the same test set, which was independent from the training procedure to test the final result of the neural network, and compared the outcomes with the reference standard.

#### Machine learning models based on clinical features

Supplementary Fig. 1 summarizes the performance of the machine learning models built on all clinical features (sex, age, clinical T-stage, clinical N-stage, adjuvant chemotherapy, and time between restaging endoscopy and surgery) and selected clinical features (clinical N-stage, adjuvant chemotherapy, and time between restaging endoscopy and surgery). The performance of models built on the three selected clinical features was higher than the model built on all clinical features. When considering the three selected clinical features, the FFN model performed slightly better (AUC of 0.73 ± 0.05; accuracy of 0.70 ± 0.04) than the SVM model (AUC 0.74 ± 0.05; accuracy 0.68 ± 0.04) and the logistic regression model (AUC 0.71 ± 0.06; accuracy 0.64 ± 0.04).

#### Deep learning models based on endoscopic images with and without clinical features

**Fig. 3:**
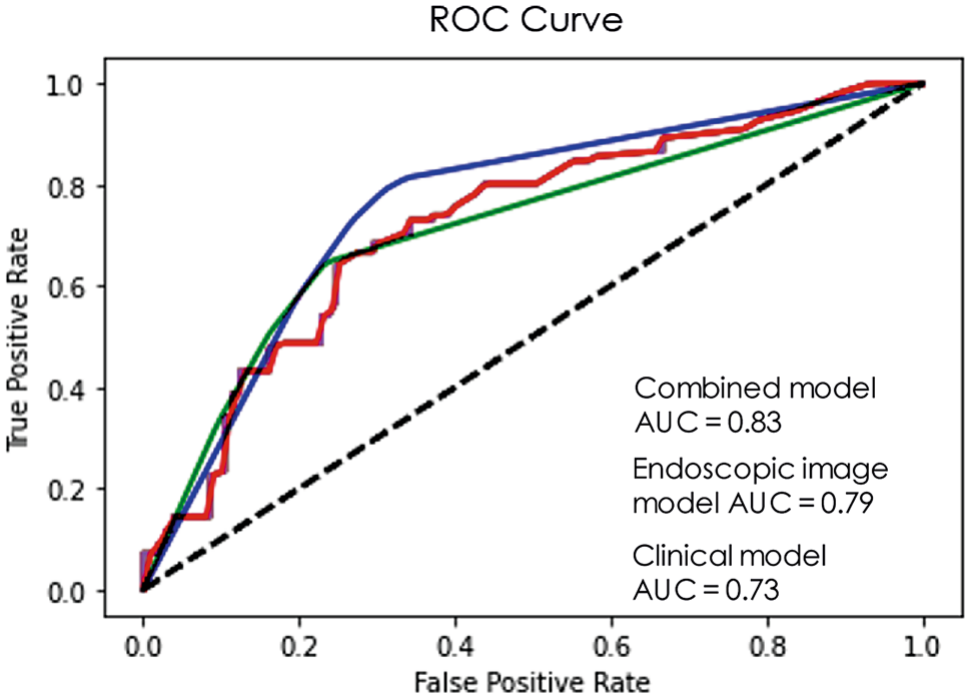
ROC curve of EfficientNet-B2 for the endoscopic image model and combined model and ROC curve of feedforward neural network model for selected clinical variables. *AUC* Area under the ROC curve

The performance of the different models using endoscopic images as an input was lower than the performance of the combined model in which imaging and clinical features were used. The AUCs for the different CNN models using endoscopic images only ranged from 0.71–0.79 and was best for EfficientNet-B2 with an AUC of 0.79 (95% CI 0.75–0.82) and a sensitivity of 0.74 (95% CI 0.70–0.78) and specificity of 0.70 (95% CI 0.66–0.74). All models based on endoscopic images only performed worse than the combined model. A detailed overview of the diagnostic performance of the endoscopic image models only are described in Supplementary Table 1. The performance of the combined models are described in Table 2. The AUCs varied from 0.76–0.83 and was the highest in EfficientNet-B2 (0.83, 95% CI 0.80–0.86). Accuracy varied from 0.67–0.75, with EfficientNet-B2 and Xception having the highest accuracy (0.75, 95% CI 0.72–0.79; and 0.75, 95% CI 0.71–0.79, respectively). The PPV was the highest using EfficientNet-B2 (0.74, 95% CI 0.70–0.77) and Xception (0.74, 95% CI 0.71–0.78) and varied from 67% to 74%. Xception had the highest NPV (0.78, 95% CI 0.74–0.80) and varied from 70% to 78%. Sensitivities varied from 68% to 79% and was the highest using Xception (0.79, 95% CI 0.75–0.82). Specificities varied between 66% and 75%, and was the highest using EfficientNet-B2 (0.75, 95% CI 0.72–0.79). Figure 3 shows the diagnostic performance for EfficientNet-B2 and Supplementary Fig. 2 presents the loss value and accuracy of the training/validation datasets. Supplementary Fig. 3 gives an overview of the misclassified patients with EfficientNet-B2.

**Table 2:** Evaluation of the different convolutional neural network models including endoscopic images and clinical variables

	Xception	MobileNet	DenseNet 121	ResNet50	InceptionV3	Inception ResNetV2	EfficientNet-B2
AUC (95% CI)	0.81 (0.78–0.84)	0.81 (0.77–0.84)	0.78 (0.74–0.82)	0.78 (0.74–0.81)	0.81 (0.77–0.84)	0.76 (0.72–80)	0.83 (0.80–0.86)
Accuracy (95% CI)	0.75 (0.71–0.79)	0.70 (0.66–0.74)	0.69 (0.65–0.73)	0.67 (0.63–0.71)	0.72 (0.68–0.76)	0.69 (0.65–0.73)	0.75 (0.72–0.79)
PPV (95% CI)	0.74 (0.71–0.78)	0.67 (0.63–0.71)	0.71 (0.67–0.74)	0.67 (0.63–0.71)	0.73 (0.69–0.77)	0.67 (0.63–0.71)	0.74 (0.70–0.77)
NPV (95% CI)	0.78 (0.74–0.80)	0.70 (0.66–0.74)	0.71 (0.67–0.75)	0.72 (0.68–0.75)	0.74 (0.70–0.77)	0.71 (0.67–0.75)	0.77 (0.74–0.80)
Sensitivity (95% CI)	0.79 (0.75–0.82)	0.76 (0.73–0.80)	0.68 (0.64–0.72)	0.73 (0.70–0.77)	0.71 (0.67–0.75)	0.68 (0.64–0.72)	0.77 (0.73–0.80)
Specificity (95% CI)	0.73 (0.69–0.77)	0.73 (0.70–0.77)	0.72 (0.68–0.76)	0.66 (0.62–0.70)	0.72 (0.69–0.76)	0.71 (0.67–0.75)	0.75 (0.72–79)

*CI* Confidence interval, *AUC* Area under the ROC curve, *PPV* Positive predictive value, *NPV* Negative predictive value

## Discussion

The present study shows a modest accuracy of deep learning models based on both endoscopic images and clinical features to detect CRs on post-CRT endoscopy, and the combined model had a higher performance than models built on clinical features or endoscopic images only. The AUCs for the different CNN models ranged from 0.76 to 0.83. The CNN models detected 68% to 79% of the patients with a luminal CR. The diagnostic performance based on endoscopic images and clinical features varied between the different models but EfficientNet-B2 achieved the highest accuracy and AUC of 0.75 (95% CI 0.72–0.79) and 0.83 (95% CI 0.80–0.86), respectively.

The AUCs in the present study are generally somewhat lower than the AUCs of 0.80 to 0.88 reported by experienced endoscopists by van der Sande et al. and Maas et al. [1, 2]. The sensitivity of the AI models lies within the range of visual evaluation by endoscopists reported in the literature: 53% to 90% [1, 2] Of course the sensitivity in the reported studies is highly dependent on the cut-off point, as illustrated by the study of Maas et al. where a sensitivity of 53% was reported when using very strict selection criteria such as a white scar without any surface irregularities as a luminal CR. Currently, in many centers the selection criteria are less strict, leading to a higher sensitivity at the expense of a lower specificity to detect a CR. The specificity among the CNN models varied from 66% to 75%, generally somewhat lower than the 61% to 97% reported by experienced endoscopists [1, 2].

Deep learning seems to be beneficial in other endoscopic areas, like in adenoma recognition where an algorithm correctly identifies diminutive (<5 mm) polyps in which a diagnose-and-leave strategy is accepted [9, 11, 31, 32]. Additionally, two randomized trials showed the efficacy of a real-time on-screen alert box in assisting endoscopists in polyp detection and evaluating the number of blind spots during procedures for quality measurements [10, 33]. In contrast to this, the current study showed a lower diagnostic value of the AI model than generally reported for expert endoscopists. Factors that may have contributed to this are the lack of high-resolution images, and the input of only a limited number of 2D images per patient. High-resolution images and real-time video assessment will likely lead to a higher performance Moreover, the algorithm in the present study calculates the probability of a CR only on the basis of the few endoscopic images, whereas experienced endoscopists can also include the information of DRE and MR-imaging [1]. An additional limitation of the study is that some patients had a long interval between endoscopy and surgery, often because patients initially refused surgery or tried an alternative treatment. This may have caused a discrepancy between endoscopic images and histology of the resection specimen.

Clinical practice is shifting from providing a W&W approach in only typical cCR (a flat white scar without any surface irregularities at the first response assessment) [12, 34] to also selecting patients with a ‘near CR’ (ulcers, irregularity or adenoma) who can develop a flat white scar at a second reassessment after another interval [35]. The definition of this so called ‘near CR’ is unclear, and the subtle abnormalities are difficult to interpret with endoscopy only [1–3]. This is an area where we hope that deep learning methods can be of help. In addition, it could help endoscopists to guide focused biopsies in doubtful cases. In clinical practice the endoscopic assessment will never serve as a single-modality for decision making. Deep learning and AI can provide the endoscopic probability of a CR, and this information has to be added to the information of the other assessment methods. The addition of DRE and MRI-DWI to endoscopic evaluation had been shown to be highly valuable, with a particular value of MRI-DWI to detect in- and extra-luminal scattered tumor regions or nodal disease [36]. Clinical decision making is a complex process, that not only involves a probability estimate, but also patient and doctor preferences, and a number of other practical and ethical issues [37, 38]. Usually, physicians refer to non-imaging clinical data to interpret endoscopic imaging findings leading to higher diagnostic accuracy and more confident clinical decisions. EfficientNet-B2 demonstrated both higher accuracy and better efficiency over existing CNNs models and they also transfer well in multiple transfer learning datasets [25]. They also performed best in our endoscopic imaging and combined models, where EfficientNet-B2 had the highest performance. In order to develop an accurate deep learning model, a large amount of data is required for collecting and labeling. However, when limited data is available, as in the current study, transfer learning has been shown to be a useful alternative, and there is evidence it even outperforms fully trained CNN models [16, 17, 39, 40]. To further explore the diagnostic performance of deep learning in response evaluation, additional studies are needed, such as multicenter cohorts evaluating a large amount of high-resolution images or video material taken by different endoscopists. Possibly, adding other clinical input (e.g., DRE and MRI findings) can further improve the models [41].

This retrospective pilot study shows that combining deep learning with clinical parameters to identify CR after neoadjuvant treatment for rectal cancer yields a diagnostic performance ranging from 0.76 to 0.83. The outcomes of CNN models varied widely, with EfficientNet-B2 being the most promising model. Compared to the literature, at present, an experienced endoscopist seems to be more accurate than deep learning. However, artificial intelligence may play a role in response evaluation when the performance of the models is further improved, and large prospective studies are required to explore this.

## Supplementary Information

Below is the link to the electronic supplementary material.

Supplementary file1 (DOCX 1773 kb)

Supplementary file2 (DOCX 15 kb)
