# Supplementary material for: The use of deep learning on endoscopic images to assess the response of rectal cancer after chemoradiation
Source: Surg Endosc. 2021 Oct 12;36(5):3592–600. doi: 10.1007/s00464-021-08685-7 (PMC9001548; doi:10.1007/s00464-021-08685-7)
Supplement: Supplementary file 1 — Supplementary file1 (DOCX 1773 kb) [file 464_2021_8685_MOESM1_ESM.docx]

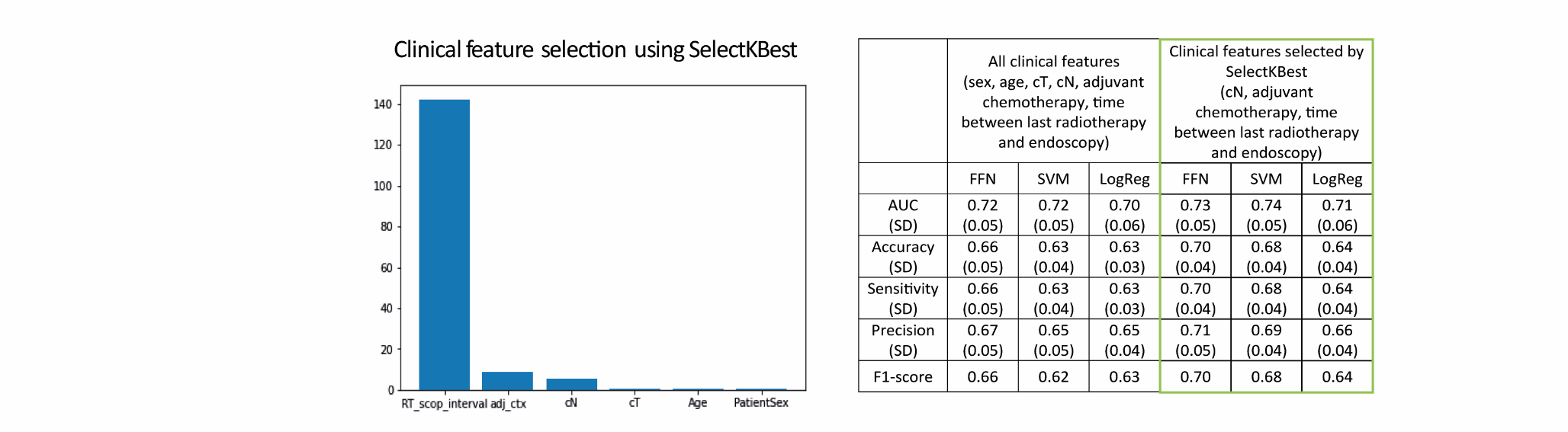


Supplementary figure 1. Clinical feature selection using SelectKBest with diagnostic performance of all the clinical features and the selected clinical features only. AUC=area under the ROC curve; SD=standard deviation; FFN=feedforward neural network; SVM=support vector machine; LogReg=logistic regression.


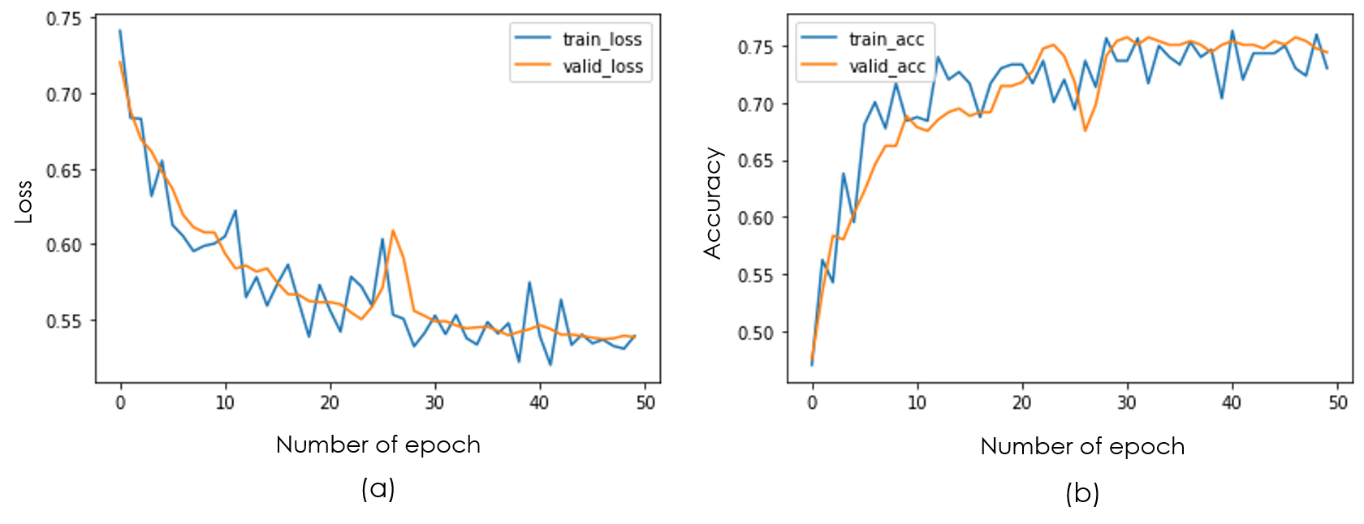


Supplementary figure 2. (a) the loss value and (b) accuracy of the training/validation dataset based on EfficientNet-B2 combined model.


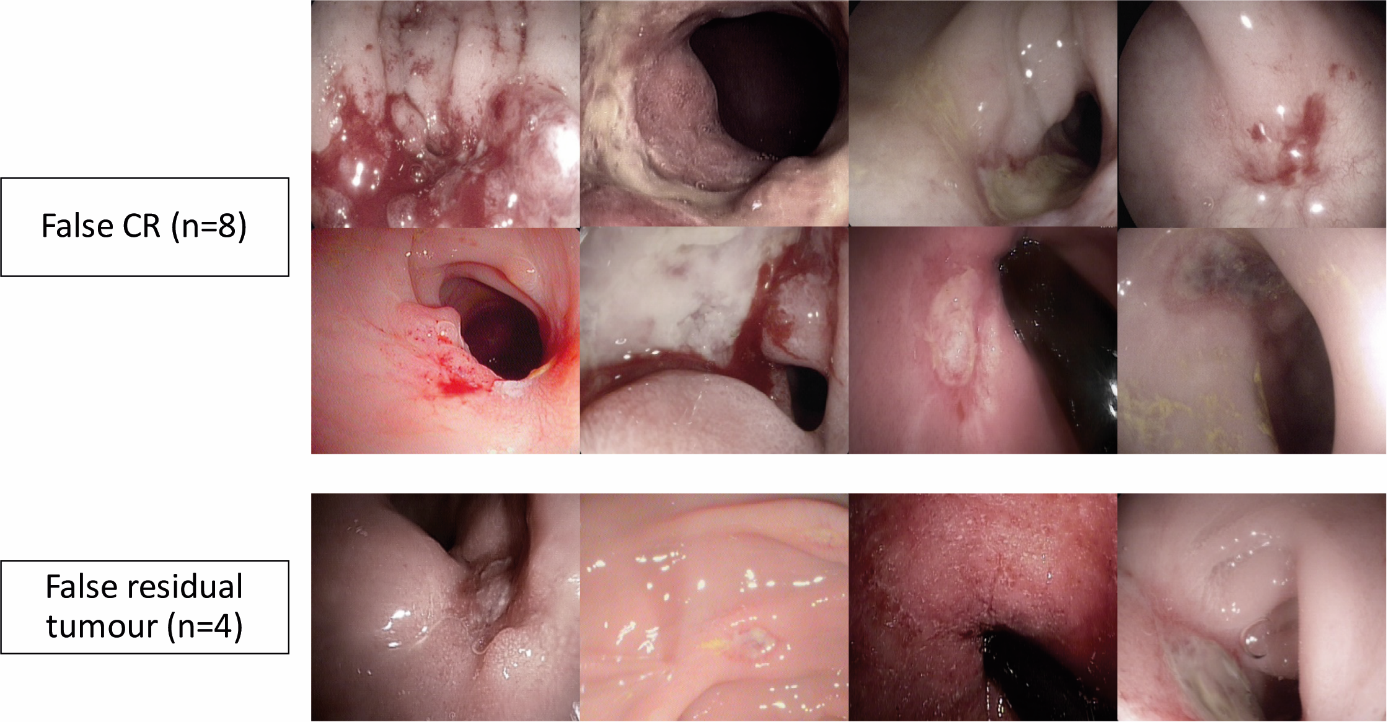


Supplementary figure 3. Misclassified images of EfficientNet-B2 for the combined model.
