# Supplementary material for: The use of deep learning on endoscopic images to assess the response of rectal cancer after chemoradiation
Source: Surg Endosc. 2021 Oct 12;36(5):3592–600. doi: 10.1007/s00464-021-08685-7 (PMC9001548; doi:10.1007/s00464-021-08685-7)
Supplement: Supplementary file 2 — Supplementary file2 (DOCX 15 kb) [file 464_2021_8685_MOESM2_ESM.docx]

|  | **Xception** | **MobileNet** | **DenseNet**  **121** | **ResNet50** | **InceptionV3** | **Inception**  **ResNetV2** | **EfficientNet-B2** |
| --- | --- | --- | --- | --- | --- | --- | --- |
| *AUC*  *(95%CI)* | 0.76  (0.73-0.80) | 0.73  (0.69-0.76) | 0.75  (0.71-0.78) | 0.71  (0.67-0.75) | 0.74  (0.70-0.77) | 0.72  (0.68-0.76) | 0.79  (0.75-0.82) |
| *Accuracy*  *(95%CI)* | 0.66  (0.62-0.70) | 0.60  (0.56-0.65) | 0.62  (0.58-0.67) | 0.63  (0.59-0.67) | 0.60  (0.56-0.64) | 0.62  (0.58-0.66) | 0.66  (0.62-0.70) |
| *PPV*  *(95%CI)* | 0.69  (0.64-0.72) | 0.65  (0.60-0.69) | 0.65  (0.61-0.70) | 0.54  (0.50-0.58) | 0.63  (0.59-0.67) | 0.66  (0.62-0.70) | 0.63  0.59-0.67) |
| *NPV*  *(95%CI)* | 0.70  (0.66-0.73) | 0.64  (0.60-0.68) | 0.66  (0.62-0.70) | 0.69  (0.65-0.73) | 0.65  (0.61-0.69) | 0.62  (0.58-0.66) | 0.68  (0.64-0.72) |
| *Sensitivity*  *(95%CI)* | 0.64  (0.60-0.68) | 0.63  (0.59-0.67) | 0.67  (0.63-0.71) | 0.68  (0.64-0.72) | 0.65  (0.61-0.69) | 0.64  (0.59-0.68) | 0.74  (0.70-0.78) |
| *Specificity*  *(95%CI)* | 0.70  (0.66-0.74) | 0.52  (0.47-0.56) | 0.62  (0.58-0.67) | 0.65  (0.61-0.70) | 0.65  (0.61-0.69) | 0.66  (0.62-0.70) | 0.70  (0.66-0.74) |

^Supplementary table 1. Evaluation of the different convolutional neural network models including endoscopic images only. CI= confidence interval; AUC=area under the ROC curve; PPV=positive predictive value; NPV=negative predictive value^
